# The complete plastome sequence of the endangered orchid *Habenaria radiata* (Orchidaceae)

**DOI:** 10.1080/23802359.2017.1390410

**Published:** 2017-10-14

**Authors:** Young-Kee Kim, Myoung Hai Kwak, Ja-Ram Hong, Hoe-Won Kim, Sangjin Jo, Jung-Yeon Sohn, Se-Hwan Cheon, Ki-Joong Kim

**Affiliations:** a Division of Life Sciences, Korea University, Seoul, Korea;; b Department of Plant Resources, National Institute of Biological Resources, Incheon, Korea

**Keywords:** *Habenaria radiata*, Orchidaceae, plastome, endangered species

## Abstract

In this study, we determined the complete chloroplast sequence of *Habenaria radiata* (Thunb.) Spreng. (Orchidaceae) (NCBI acc. no. KX871237), an endangered plant species protected by the national law of Korea. The gene order and gene content of the *H. radiata* plastome are similar to those of typical angiosperm plastomes. The 11 *ndh* genes, which are usually lost in orchid plastomes, are intact in the *H. radiata* plastome. The complete plastome is 155,353 bp in length and consists of a large single copy of 84,833 bp and a small single copy of 17,718 bp, separated by two inverted repeats of 26,401 bp. The plastome contains 113 genes, of which 79 are protein-coding genes, 30 are tRNA genes, and four are rRNA genes. Sixteen genes contain one intron and two genes (*clp*P, *ycf*3) have two introns. A total of 76 simple sequence repeat (SSR) loci, which consist of 58 mono-SSR, 17 di-SSR, and 1 tri-SSR, are scattered along the *H. radiata* plastome. Some of these plastome SSR and high sequence divergent regions may be useful for development of genetic markers for the populations of *H. radiata* and other congeneric species. Phylogenetic analysis identified the sister relationship between *H. radiata* and *H. pantlingiana* within the tribe Orchideae.

*Habenaria radiata* (Thunb.) Spreng., one of the terrestrial orchids in the genus *Habenaria*, is native to Korea and Japan (Lee 2011). It is a single-stemmed, erect plant, up to 40 cm tall, with small root tubers. *H. radiata* was relatively common in moisture-rich areas on mountain slopes or in grasslands until a few decades ago. The species has been often targeted by orchid collectors and hobbyist because of the beautiful bird-like floral structure. As a result, the number of natural populations of *H. radiata* has decreased rapidly in recent years. Only a few populations have been sporadically reported from the remote mountain areas of Korea. Therefore, the species was designated as endangered species, and it was protected by the national law of Korea. The genus *Habenaria*, comprising about 835 species (Chase et al. 2015), belongs to the subfamily Orchidoideae of the family Orchidaceae in the order Asparagales (APG IV 2016). In order to develop genetic markers of *H. radiata* for conservation studies, we sequenced and analysed the plastome of this species. The plastome data will be useful to evaluate the genetic diversity and the phylogenetic position of *H. radiata* and related taxa.

The seeds of *H. radiata* were originally collected from a natural population in the National Arboretum of Korea. The leaf material of *H. radiata* was collected from a single individual that was cultivated in a laboratory flask from the seeds. A voucher specimen and DNA sample were deposited in the Korea University Herbarium (KUS 2015-1271) and the Plant DNA Bank in Korea (PDBK 2015-1271), respectively. Fresh leaves were ground into powder in liquid nitrogen, and total DNA was extracted by using G-spin™II for Plant Genomic DNA extraction kit (iNtRON, Seongnam, Korea). The complete plastome was generated using Illumina MiSeq (San Diego, CA), and assembled with Geneious 6.1.8 (Kearse et al. 2012). The average coverage of the sequence was 962 times that of the plastome size. Annotations were performed using the National Center for Biotechnology Information (NCBI) BLAST and tRNAscan-SE programs (Lowe and Eddy 1997). The complete plastome sequence was submitted to the NCBI database under accession number KX871237.

The gene order and gene content of the *H. radiata* were similar to those of a typical angiosperm such as *Panax, Nicotina*, and *Sesamum* (Shinozaki et al. 1986; Kim and Lee 2004; Yi and Kim 2012). The orchid plastomes usually lost the *ndh* genes (Chang et al. 2006; Wu et al. 2010; Lin et al. 2015). However, the *H. radiata* plastome retained all of the *ndh* genes. The plastome of *H. radiata* contains only 113 unique genes, including 79 protein-coding genes, 30 tRNA genes, and four rRNA genes. Sixteen genes had a single intron, whereas the *clp*P and *ycf*3 genes had two introns. The length of the complete plastome of *H. radiata* was 155,353 bp, and it was composed of a large single copy (LSC) of 84,833 bp, a small single copy (SSC) of 17,718 bp, and two inverted repeats (IRs) each 26,401 bp long. The length of the *H. radiata* plastome was approximately 10 kb longer than that of the typical orchid plastome. The average AT content of the plastome was 63.5%. We identified a total of 76 simple sequence repeat (SSR) loci, which consisted of 58 mono-SSR, 17 di-SSR, and 1 tri-SSR, scattered along the plastome. Some of these plastome SSRs may be useful for development of genetic markers for the populations of *H. radiata*.

For phylogenetic tree construction, 82 gene sequences from 44 taxa were aligned using MUSCLE implemented in Geneious 6.1.8. The aligned data matrix was 70,020 bp in length. The data matrix was subjected to phylogenetic analysis using RAxML v. 7.7.1 (Stamatakis et al. 2008). An ML tree was obtained with an ML optimization likelihood value of –314504.317667. A sister group relationship between the two *Habenaria* species was supported by 100% bootstrap support (Figure 1). In order to clarify more precisely the phylogenetic relationships of *Habenaria* and other Orchidaceae members, we need additional whole plastome data from diverse genera and species of the tribe Orchideae.

**Figure 1.**
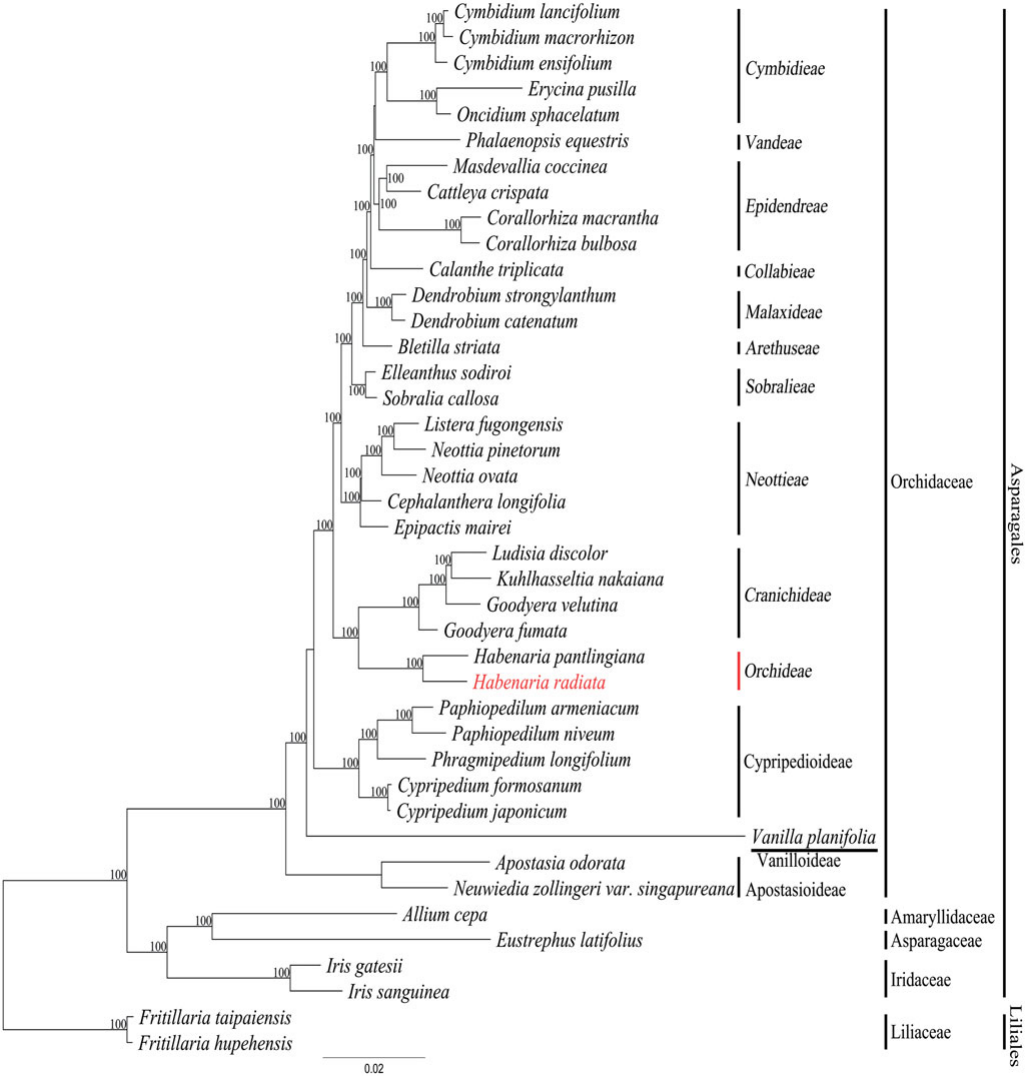
A maximum likelihood (ML) tree of Orchidaceae indicating the phylogenetic position of *Habenaria radiata*. The ML tree is reconstructed from the sequences of 78 protein coding genes and four rRNA genes for 41 plastome sequences. The number above or below of each node indicates bootstrap supporting value from 100 replications. Genbank accession numbers of taxa are shown below: *Allium cepa* (NC024813), *Apostasia odorata* (NC030722), *Bletilla striata* (NC028422), *Calanthe triplicata* (NC024544), *Cattleya crispata* (NC026568), *Cephalanthera longifolia* (NC030704), *Corallorhiza bulbosa* (NC025659), *Corallorhiza macrantha* (NC025660), *Cymbidium ensifolium* (NC028525), *Cymbidium lancifolium* (NC029712), *Cymbidium macrorhizon* (KY354040), *Cypripedium formosanum* (NC026772), *Cypripedium japonicum* (NC027227), *Dendrobium catenatum* (NC024019), *Dendrobium strongylanthum* (NC027691), *Elleanthus sodiroi* (NC027266), *Epipactis mairei* (NC030705), *Erycina pusilla* (NC018114), *Eustrephus latifolius* (NC025305), *Fritillaria hupehensis* (NC024736), *Fritillaria taipaiensis* (NC023247), *Goodyera fumata* (NC026773), *Goodyera velutina* (NC029365), *Habenaria radiata* (KX871237), *Habenaria pantlingiana* (NC026775), *Iris gatesii* (NC024936), *Iris sanguinea* (NC029227), *Kuhlhasseltia nakaiana* (KY354041), *Listera fugongensis* (NC030711), *Ludisia discolor* (NC030540), *Masdevallia coccinea* (NC026541), *Neottia ovata* (NC030712), *Neottia pinetorum* (NC030710), *Neuwiedia zollingeri var. singapureana* (KM244735), *Oncidium sphacelatum* (NC028148), *Paphiopedilum armeniacum* (NC026779), *Paphiopedilum niveum* (NC026776), *Phalaenopsis equestris* (NC017609), *Phragmipedium longifolium* (NC028149), *Sobralia callosa* (NC028147), *Vanilla planifolia* (NC026778).
